# LAGE3 promoted cell proliferation, migration, and invasion and inhibited cell apoptosis of hepatocellular carcinoma by facilitating the JNK and ERK signaling pathway

**DOI:** 10.1186/s11658-021-00295-4

**Published:** 2021-11-27

**Authors:** Ying Xing, Yang Liu, Zhong Qi, Zhengrong Liu, Xin Wang, Hongyi Zhang

**Affiliations:** Department of General Surgery, Beijing Tiantan Hospital, Capital Medical University, No. 119, South 4th Ring West Road, Fengtai District, Beijing, 100070 China

**Keywords:** Hepatocellular carcinoma, L antigen family member 3, Malignant phenotypes, JNK and ERK signaling pathway

## Abstract

**Background:**

Hepatocellular carcinoma (HCC) is now the second leading cause of cancer death worldwide and lacks effectual therapy due to its high rate of tumor recurrence and metastasis. The aim of this study was to investigate the effects of L antigen family member 3 (LAGE3, a member of the LAGE gene family involved in positive transcription) on the progression of HCC.

**Methods:**

The expression of LAGE3 was detected by quantitative real-time polymerase chain reaction. 3-(4,5-dimethylthiazol-2-yl)-2,5-diphenyltetrazolium bromide, colony formation assay, EdU, and cell cycle analysis assay were employed to evaluate the proliferation of HCC cells. Annexin V-FITC/PI and TUNEL assay were used to assess the apoptosis rate of HCC cells. Wound healing and transwell assay were used to investigate the migration and invasion of HCC cells. A xenograft model of HCC was established with 2 × 10^6^ Hep3B or SK-HEP1 cells to investigate the in vivo effects of LAGE3. Then, the protein levels of LAGE3, p-p38, p-38, c-Jun N-terminal kinase (JNK),p-JNK, extracellular signal-regulated kinase (ERK), and p-ERK were detected by western blot.

**Results:**

We found that LAGE3 was upregulated in HCC tissues compared to adjacent tissues, and its high expression was correlated with poor overall survival by bioinformatics analysis. Next, we manually regulated the expression of LAGE3 in HCC cells. The knockdown of LAGE3 inhibited the proliferation of HCC cells by arresting the cell cycle in G1 phase. Also the downregulation of LAGE3 inhibited cell migration and invasion and induced apoptosis of HCC cells, while overexpression of LAGE3 promoted the malignant phenotypes of HCC. These results were further confirmed by the in vivo growth of HCC xenografts and the inhibition of apoptosis of HCC tumor cells. Furthermore, we found that LAGE3 exerted cancer-promoting effects by potentiating the JNK and ERK signaling pathway. An ERK inhibitor (10 μM SCH772984) or JNK inhibitor (25 μM SP600125) repressed the upregulated LAGE3-induced proliferation, migration, and invasion of HCC cells.

**Conclusions:**

LAGE3 enhanced the malignant phenotypes of HCC by promoting the JNK and ERK signaling pathway.

## Background

Hepatocellular carcinoma (HCC) accounts for approximately 85–90% of all cases of primary liver cancer and is now the second leading cause of cancer death worldwide, with over 780,000 annual deaths globally in recent years [1–3]. HCC is common in patients with unhealthy alcohol intake, non-alcoholic fatty liver disease, infection with hepatitis B and C, as well as advanced hepatic fibrosis or cirrhosis [4]. They promote hepatic inflammation and aberrant hepatocyte regeneration, leading to a series of genetic and epigenetic events. These events provide dysplastic tumor cells with proliferative, migrative, invasive, and survival advantages and achieve the transition to the advanced stage of HCC [5, 6]. Currently, surgical treatment is the most effective therapy of HCC only for patients in the early stage of the disease, but about 60–70% of patients are not diagnosed until late stages [7]. In this case, the efficacy of radiotherapy and chemotherapy is limited [7]. The high rates of tumor recurrence and metastasis are the main factors that contribute to the poor prognosis and the low five-year survival rate of HCC [8]. Thus, there is an urgent need to deeply investigate the mechanism of HCC and explore effectual molecules targeting the treatment of this fatal malignancy.

L antigen family member 3 (LAGE3, NM_006014), a member of the LAGE gene family, is ubiquitously expressed in somatic tissues in humans [9]. LAGE3 is also considered a component of the kinase, endopeptidase and other proteins of small size/endopeptidase-like kinase chromatin-associated protein complex, playing a vital role in the regulation of the positive transcription mediated by RNA polymerase II, tRNA metabolic processing and ncRNA processing [10, 11]. The mutation of LAGE3 is linked with nephrotic syndrome associated with primary microcephaly, brain abnormalities and growth retardation [12]. Accumulating evidence has shown that LAGE3 is recognized as an overexpressed RNA modification-related protein in many cancers [13]. Dong et al. found that LAGE3 is highly expressed in papillary thyroid cancer and is associated with advanced malignancy, biochemical metabolism, and immune-related terms including immune cells infiltrating levels and the activity of different steps of the cancer-immunity cycle [14]. The overexpression of LAGE3 has been reported to correlate with the poor prognosis of clear cell renal cell carcinoma and independently determine the survival of patients [15]. LAGE3 is a potential therapeutic target of breast cancer, and its upregulation is linked with the aggressive progression of breast cancer [16, 17]. Based on these essential functions of LAGE3 in malignant phenotypes of cancer, we considered whether LAGE3 could play a role in the progression of HCC. However, its distinct function of HCC has not been reported.

In this study, we aimed to investigate the effects of LAGE3 on the progression of HCC. We found that LAGE3 was upregulated in HCC tissues compared to adjacent tissues. Its high expression was associated with poor overall survival of HCC by bioinformatics analysis. We manually regulated the expression of LAGE3 in HCC cells and examined its effects on cell proliferation, migration, invasion, and apoptosis in HCC. Then, a xenograft model of HCC was established to assess the in vivo function of LAGE3. Additionally, the underlying mechanism of LAGE3 in HCC pathogenesis was further explored. Our research indicated that the downregulated LAGE3 might serve as a potential target for the effective strategy of HCC treatment.

## Methods

### Cell culture, transfection, and treatment

Huh7, Hep3B, SK-HEP1, and HCC-LM3 cells were purchased from Procell (Wuhan, China). Hep3B cells were purchased from iCell Bioscience Inc. (Shanghai, China). All cell lines were authenticated by a short tandem repeat profiled every year, and a mycoplasma test was carried out every three months. Huh7 and HCC-LM3 cells were cultured with DMEM medium supplemented with 10% fetal bovine serum (FBS). Hep3B and SK-HEP-1 cells were cultured in MEM medium supplemented with 10% FBS. All cells were cultured within a 5% CO_2_ atmosphere at 37 °C.

For the overexpression of LAGE3, the cDNA of LAGE3 was amplified and inserted into a pcDNA3.1 (Clontech, Mountain View, CA, USA) vector. Short interfering RNAs (siRNAs) targeting LAGE3 were designed to construct LAGE3-depleted HCC cells. At the same time, the short hairpin RNA (shRNA) for LAGE3 was subcloned into a pRNA-H1.1 plasmid (GenScript, Nanjing, China). Transient transfections were performed in Hep3B or SK-HEP1 cells by using Lipofectamine 3000 (Invitrogen, Carlsbad, California, USA) following the users’ instructions.

After transfected for 48 h, LAGE3-upregulated Hep3B cells were incubated with 10 μM ERK inhibitor SCH772984 (MCE, Monmouth Junction, NJ, USA) or 25 μM JNK inhibitor SP600125 (Aladdin, Shanghai, China) for 24 h at 37 °C.

### Quantitative real-time polymerase chain reaction (qRT-PCR)

Total RNA was extracted from the transfected cells using an RNAsimple Total RNA kit (Tiangen Biotech, Beijing, China), and its reverse transcription was performed by employing M-MLV reverse transcriptase (Tiangen) as per the manufacturer’s protocol. The amplification was achieved by using 2 × Taq PCR MasterMix (Tiangen) in the presence of SYBR Green (Solarbio, Beijing, China). Primers were synthesized by GenScript (Nanjing, China), and the sequences were as follows. LAGE3 F: 5ʹ-CACCCTCAGCGTGCCTTTC-3’, LAGE3 R: 5’-GATCCTTCCCAACCACCCTTT-3’; GAPDH F: 5’-GACCTGACCTGCCGTCTAG-3’, GAPDH R: 5’-AGGAGTGGGTGTCGCTGT-3’.

### Western blot

Cell protein extracts were prepared with 1 mM phenylmethanesulfonyl fluoride (diluted 1:100 with lysis solution by volume; Solarbio). The concentration of the protein was detected by the BCA Protein Assay kit (Solarbio). 20 μg of protein was subjected to sodium dodecyl sulfate polyacrylamide gel electrophoresis, and blotted onto polyvinylidene fluoride membrane (Millipore, Billerica, MA, USA). After blocking with 5% skimmed milk, blots were incubated with primary antibodies (diluted 1:3000; Solarbio) at 4 °C overnight. Next, blots were developed using horseradish peroxidase-conjugated secondary antibodies at 37 °C for an hour and the electrochemiluminescence detection reagent (Solarbio) according to the users’ instructions. Chemiluminescence signals were captured with the WD-3413B imaging system (Liuyi Biotech, Beijing, China). All used primary antibodies were as follows: anti-LAGE3 (diluted 1:1000; Biorbyt, Cambridge, Cambridgeshire, England), p38 (diluted 1:1000; Affinity, Changzhou, China), p-p38 (diluted 1:1000; Affinity), c-Jun N-terminal kinase (JNK; diluted 1:1000; Affinity), p-JNK (diluted 1:1000; Affinity), extracellular signal-regulated kinase (ERK, diluted 1:1000; Affinity), p-ERK (diluted 1:1000; Affinity), and GAPDH (diluted 1:10000; Proteintech, Wuhan, China).

### 3-(4,5-dimethylthiazol-2-yl)-2,5-diphenyltetrazolium bromide (MTT) assay

4 × 10^3^ cells per well were plated into 96-well plates. After transfected for 48 h, cells were cultured for 0 h, 24 h, 48 h, 72 h, or 96 h at 37 °C. Next, cells were maintained in completed medium supplemented with 0.5 mg/mL MTT solution (Keygen Biotech, Nanjing, China) at 37 °C for 4 h, and then 150 μL of dimethyl sulfoxide solution (Beyotime, Shanghai, China) was added to the cells for 10 min in the dark. The OD value was measured by an 800TS Microplate Reader (BioTek, Winooski, VT, USA) at a wavelength of 570 nm.

### Colony formation assay

Transfected cells were seeded into 35 mm cell culture dishes (400 cells per dish) and then cultured at 37 °C for approximately two weeks. Next, cells were washed twice with phosphate buffer saline (PBS) and fixed with 4% paraformaldehyde at room temperature for 20 min. After washing with PBS, cells were stained with Wright-Giemsa staining solution (Keygen Biotech) for 5 min. The colonies containing more than 50 cells were counted. The colony formation rate was calculated as follows: number of colonies/number of plated cells * 100%.

### EdU proliferation assay

Cell proliferation was measured by employing a kFluro488 Click-iT EdU kit (Keygen Biotech) according to the manufacturer’s instructions. In brief, 4 × 10^3^ cells per well were plated into 96-well plates. After transfection for 48 h, cells were treated with 10 μM EdU for 2 h at 37 °C and then fixed with 4% paraformaldehyde for 15 min at room temperature. After washing with PBS in 3% bovine serum albumin twice, cells were permeabilized with 0.1 mL of 0.5% Triton X-100 for 20 min at room temperature and incubated with Click-iT solution for 30 min, in absence of light. Then, cells were incubated with Hoechst 33342 solution (diluted 1:2000) for 15 min. The percentage of EdU-stained positive cells was imaged with an IX53 microscope (Olympus, Tokyo, Japan).

### Cell cycle analysis

We evaluated the changes of cell cycle using a Cell Cycle and Apoptosis Analysis Kit (Beyotime) in accordance with the users’ instructions. In brief, transfected cells were collected and washed with PBS twice. Next, cells were fixed with 70% ice-cold ethanol at 4 °C for 2 h. After centrifugation at 1000 g for 5 min, cells were incubated with 25 μL of propidium iodide (PI) and 10 μL of RNase A at 37 °C for 30 min in the dark. Then, cells were subjected to fluorescence activated cell sorting measurement. The cell cycle analysis was performed using NovoCyte flow cytometry (ACEA Biosciences, San Diego, California, USA).

### Annexin V/PI assay

We quantified the early apoptosis rate of HCC cells by employing an Annexin V-fluorescein isothiocyanate (FITC)/PI staining kit (Keygen) following the manufacturer’s protocol. In brief, after centrifugation, transfected cells were suspended with 500 μL of binding buffer and incubated with 5 μL of Annexin V-FITC and 5 μL of PI for 10 min at room temperature, in absence of light. The percentage of early apoptosis cells was detected by NovoCyte flow cytometry.

### TUNEL assay

Cell climbing sheets were fixed with 200 μL of 0.1%Trixon X-100 for 15 min (50 μL of 0.1%Trixon X-100 for 8 min for tumor sections) at room temperature and incubated with 200 μL (50 μL for tumor sections) of TUNEL solution (enzyme solution was diluted with label solution at 1:9 for volume) for 1 h in the dark. After rinsing with PBS, cell climbing sheets and tumor sections were counterstained with DAPI for 5 min. The images were captured with a BX53 microscope (Olympus).

### Xenograft model of HCC and plasmid treatment

The protocols of animal experiments were approved by the Institutional Review Board of Beijing Tiantan Hospital, Capital Medical University following the Guideline for the Care and Use of Laboratory Animals. Six-week-old female BALB/c nude mice were purchased from HFK Bioscience (Beijing, China). The establishment of the xenograft model and the plasmid treatment were performed as described previously [18]. Mice were placed in a 12-h-light/dark cycle with food and water ad libitum. After one week of acclimation, 20 mice were randomly divided into four groups (Vector, LAGE3-OE, NC shRNA, and LAGE3 shRNA). Mice were subcutaneously injected with 2 × 10^6^ HCC827 or SK-HEP1 cells. When the tumor grew to 50 mm^3^, mice received a tail-vein injection with 10 μg of LAGE3-OE or LAGE3 shRNA plasmid three times a week for four weeks. The tumor diameters were measured every four days to calculate the tumor volume. After 32 days, mice were sacrificed, and the tumors were collected for further experiments.

### Immunohistochemistry

Paraffin-embedded sections of xenograft tumors from the nude mice (5 μm) were deparaffinized in xylene, and rehydrated in an ethanol gradient with distilled water. Then, sections were incubated with 3%H_2_O_2_ for 15 min at room temperature. After blocking with 1% bovine serum albumin, sections were incubated with anti-LAGE3 (diluted 1:100; Novus Biologicals, Littleton, CO, USA) or anti-ki67 (ABclonal, Wuhan, China) at 4 °C overnight, followed by incubation with horseradish peroxidase-conjugated secondary antibodies (diluted 1:100; Thermo Fisher Scientific, Pittsburgh, PA, USA) for an hour at 37 °C. After development with 100 μL of 3,3ʹ-diaminobenzidine reagent, sections were counterstained with hematoxylin for 3 min. Images were captured by a BX53 microscope.

### Wound healing assay

Cells were seeded into 6-well plates and grown to 80% confluence. After transfection for 24 h, cells were wounded by scratching with 200 μL pipette tips. Then, cells were rinsed with serum-free medium to remove the exfoliated cells and cultured for 24 h at 37 °C. The relative wound width was imaged by an IX53 microscope at each time point.

### Transwell assay

Cells (2 × 10^4^ cells per well) were plated into the upper chambers of the transwell inserts (Corning, NY, USA) pre-coated with 40 μL of Matrigel (Corning) and maintained in serum-free medium. Medium supplemented with 10% FBS was added to the lower chambers, and cells were cultured for 24 h at 37 °C. Subsequently, invaded cells were fixed with 4% paraformaldehyde (Aladdin, Shanghai, China) for 25 min at room temperature and stained with 0.4% crystal violet (Amresco, Shanghai, China) for 5 min. Images were captured with an IX53 microscope, and five random fields were selected to count the number of invasive cells.

### Statistical analysis

Statistical analysis was carried out using GraphPad Prism 8.0 software. Differences among more than two groups were measured by one-way analysis of variance or two-way analysis of variance, and the comparison between two groups was conducted using the t-test. Data are shown as mean ± standard deviation from at least three independent experiments. p < 0.05 was considered statistically significant.

## Results

### LAGE3 was upregulated in HCC tissues and correlated with poor overall survival

**Fig. 1:**
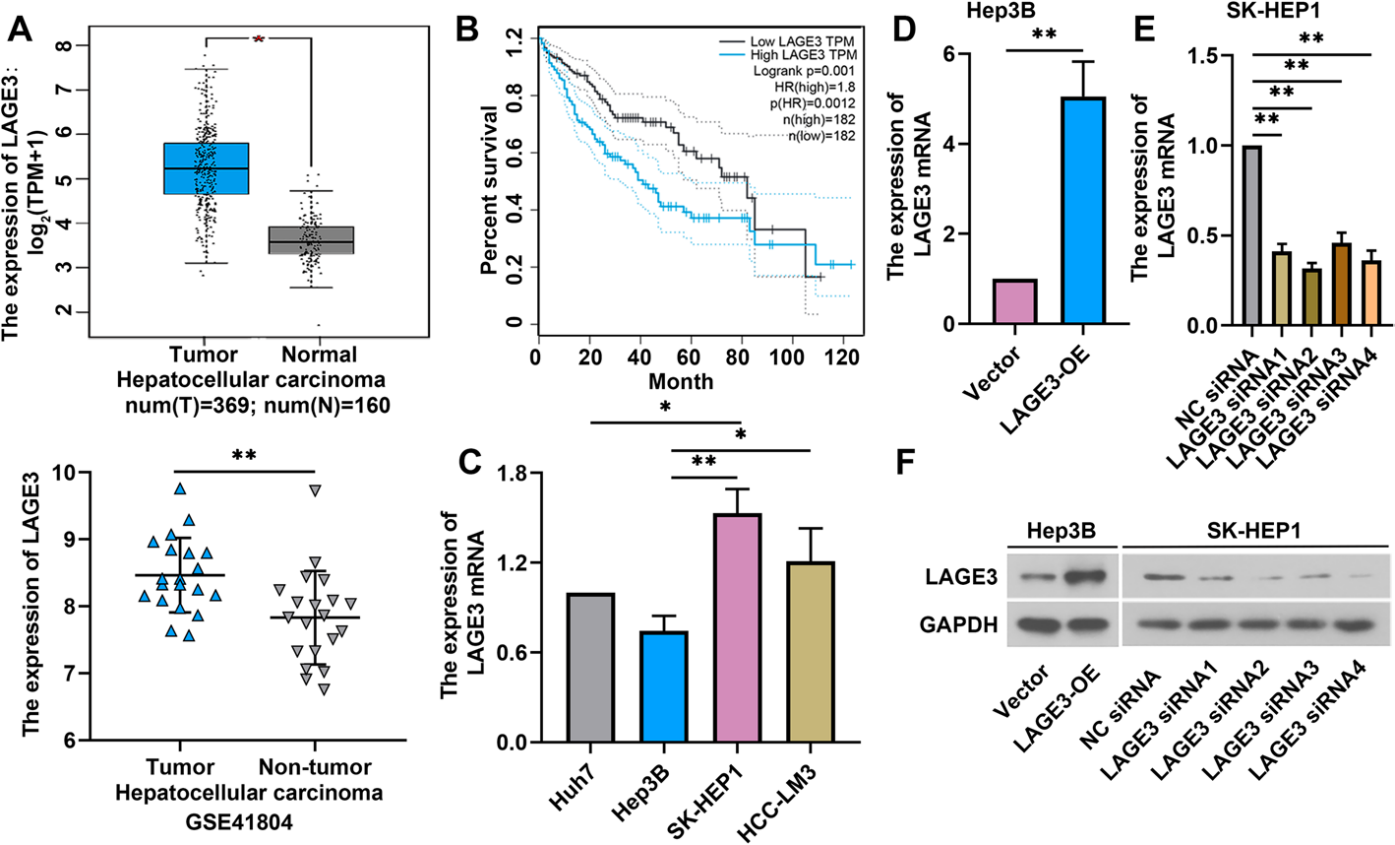
LAGE3 was upregulated in HCC and correlated with poor overall survival. **A** LAGE3 expression in HCC. TPM, transcripts per million. **B** Overall survival of HCC patients with high LAGE3 expression. HR, hazard ratio. **C** Expression of LAGE3 mRNA in four HCC cell lines (Huh7, Hep3B, SK-HEP1, and HCC-LM3 cells). **D** Relative expression of LAGE3 in Hep3B cells transfected with LAGE3-OE. **E** Effects of four different siRNA sequences targeting LAGE3 on its gene expression in SK-HEP1 cells. **F** Western blot bands of LAGE3 in Hep3B cells transfected with LAGE3-OE or SK-HEP1 cells transfected with LAGE3 siRNAs. Data are shown as mean ± standard deviation. *p < 0.05, **p < 0.01

We first compared the different expression of LAGE3 from the HCC and the normal tissues in the Cancer Genome Atlas and Gene Expression Omnibus database. As shown in Fig. 1A, LAGE3 was upregulated in HCC tissues compared to normal tissues. Patients with high expression of LAGE3 had shorter overall survival (Fig. 1B). Next, we detected the levels of LAGE3 in HCC cell lines: Huh7, Hep3B, SK-HEP1, and HCC-LM3 cells. The expression of LAGE3 was the highest in SK-HEP1 cells and the lowest in Hep3B cells (Fig. 1C). Thus, to assess the relation between LAGE3 levels and the progression of HCC cells, we manually regulated the expression of LAGE3. LAGE3 was highly expressed in Hep3B cells transfected with LAGE3-OE and showed low expression in SK-HEP1 cells transfected with LAGE3 siRNAs (Fig. 1D–E). Two siRNA sequences targeting LAGE3 expression with the highest transfection efficiency were chosen for the subsequent studies. The changes of LAGE3 protein level were consistent with the results of qRT-PCR in Hep3B and SK-HEP1 cells (Fig. 1F).

### LAGE3 promoted proliferation of HCC cells

**Fig. 2:**
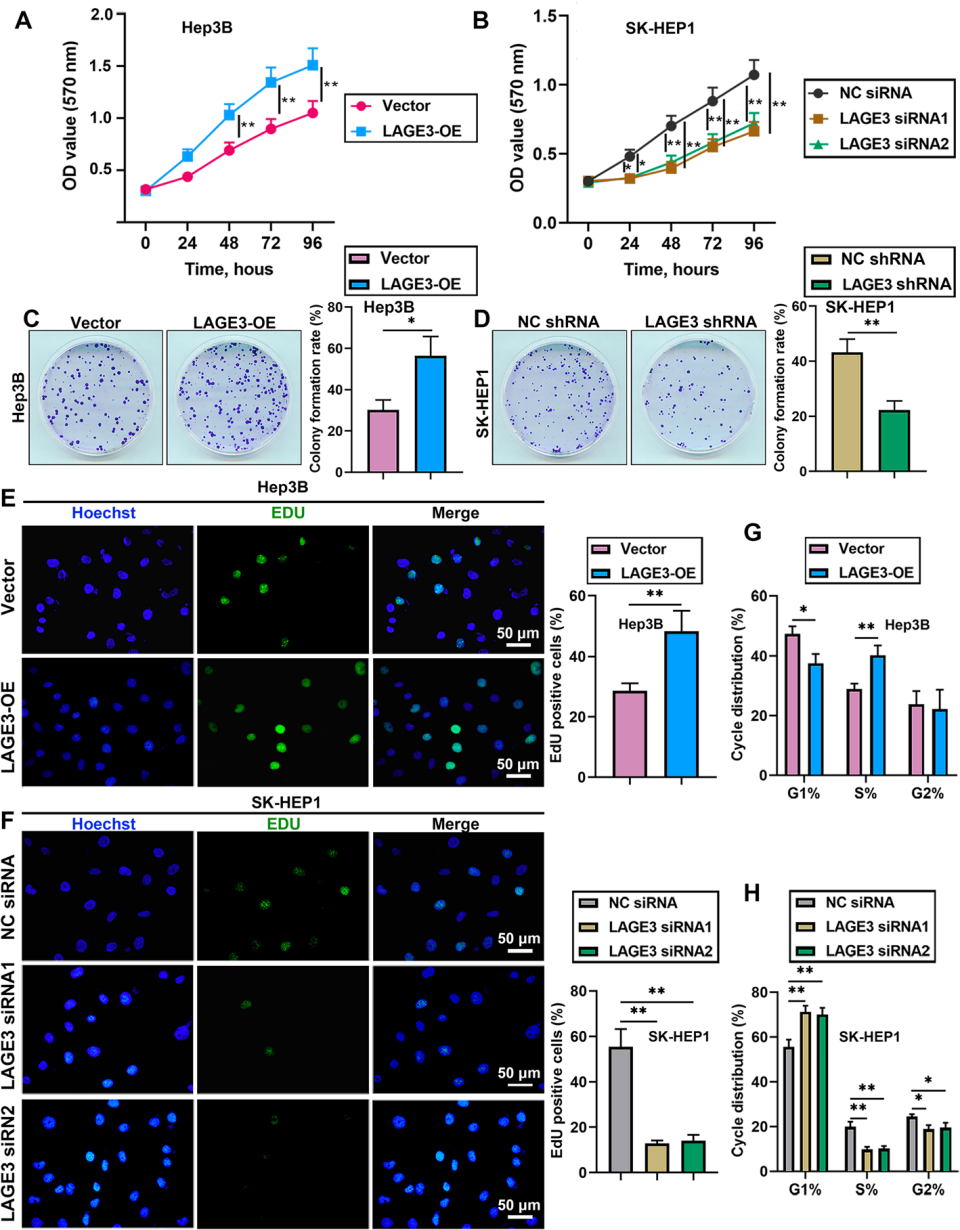
LAGE3 promoted the proliferation of HCC cells. Hep-3B cells were transfected with LAGE3-OE and SK-HEP1 cells were transfected with LAGE3 siRNA1/2 or LAGE3 shRNA. **A**–**B** MTT assay was used to evaluate the proliferation of Hep-3B and SK-HEP1 cells. **C**–**D** Colony formation assay was employed to assess the colony formation ability of Hep-3B and SK-HEP1 cells. **E**–**F** EdU-stained HCC cells. **G–H** Cell cycle analysis of HCC cells. Data are shown as mean ± standard deviation. *p < 0.05, **p < 0.01

To investigate the role of LAGE3 in HCC progression, we evaluated the effects of LAGE3 on cell proliferation by employing the MTT assay. As revealed in Fig. 2A and B, the overexpressed LAGE3 promoted the proliferation of Hep3B cells and the knockdown of LAGE3 inhibited the proliferation of SK-HEP1 cells. Next, we found that the colony formation ability was facilitated by LAGE3 upregulation and was attenuated by LAGE3 downregulation (Fig. 2C and D). Similar results of proliferation were observed by using the EdU proliferation assay (Fig. 2E and F). Moreover, the results of cell cycle analysis demonstrated that overexpression of LAGE3 resulted in a decrease in the percentage of G1 phase cells accompanied by an increase in S phase (Fig. 2G). The downregulation of LAGE3 led to an elevated percentage of the G1 phase cells with a reduced percentage of S phase and G2 phase cells (Fig. 2H). These results indicated that LAGE3 promoted the proliferation of HCC cells by enhancing the transition of G1/S phases.

### LAGE3 repressed apoptosis of HCC cells

**Fig. 3:**
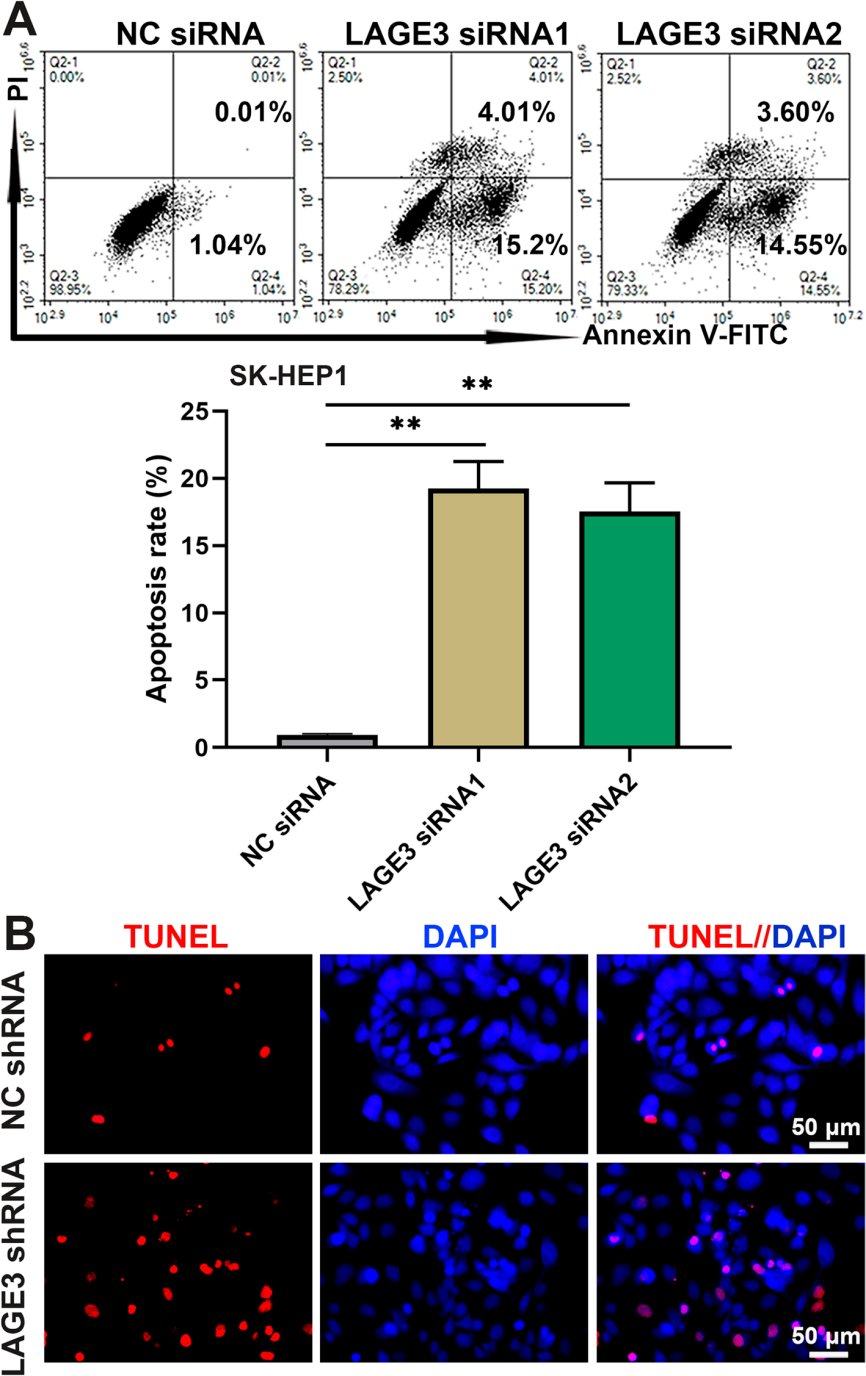
LAGE3 repressed the apoptosis of HCC cells. SK-HEP1 cells were transfected with LAGE3 siRNA1/2 or LAGE3 shRNA. **A** Annexin V-FITC/PI labeled cells were analyzed by flow cytometry to determine the early apoptosis rate. **B** TUNEL staining of late apoptotic cells. Data are shown as mean ± standard deviation. **p < 0.01

We further assessed the effects of LAGE3 on the early apoptosis rate of HCC cells by using the Annexin V-PI flow cytometry staining assay. The results showed that interfering in LAGE3 expression with siRNAs increased the early apoptosis index of SK-HEP1 cells (Fig. 3A). The results of TUNEL assay indicated that the late apoptosis rate of LAGE3-downregulated SK-HEP1 cells was the same as the changes of early apoptosis in SK-HEP1 cells (Fig. 3B). The data suggested that LAGE3 repressed the apoptosis of HCC cells.

### *LAGE3 promoted *in vivo* growth and suppressed apoptosis of HCC xenograft tumors*

**Fig. 4:**
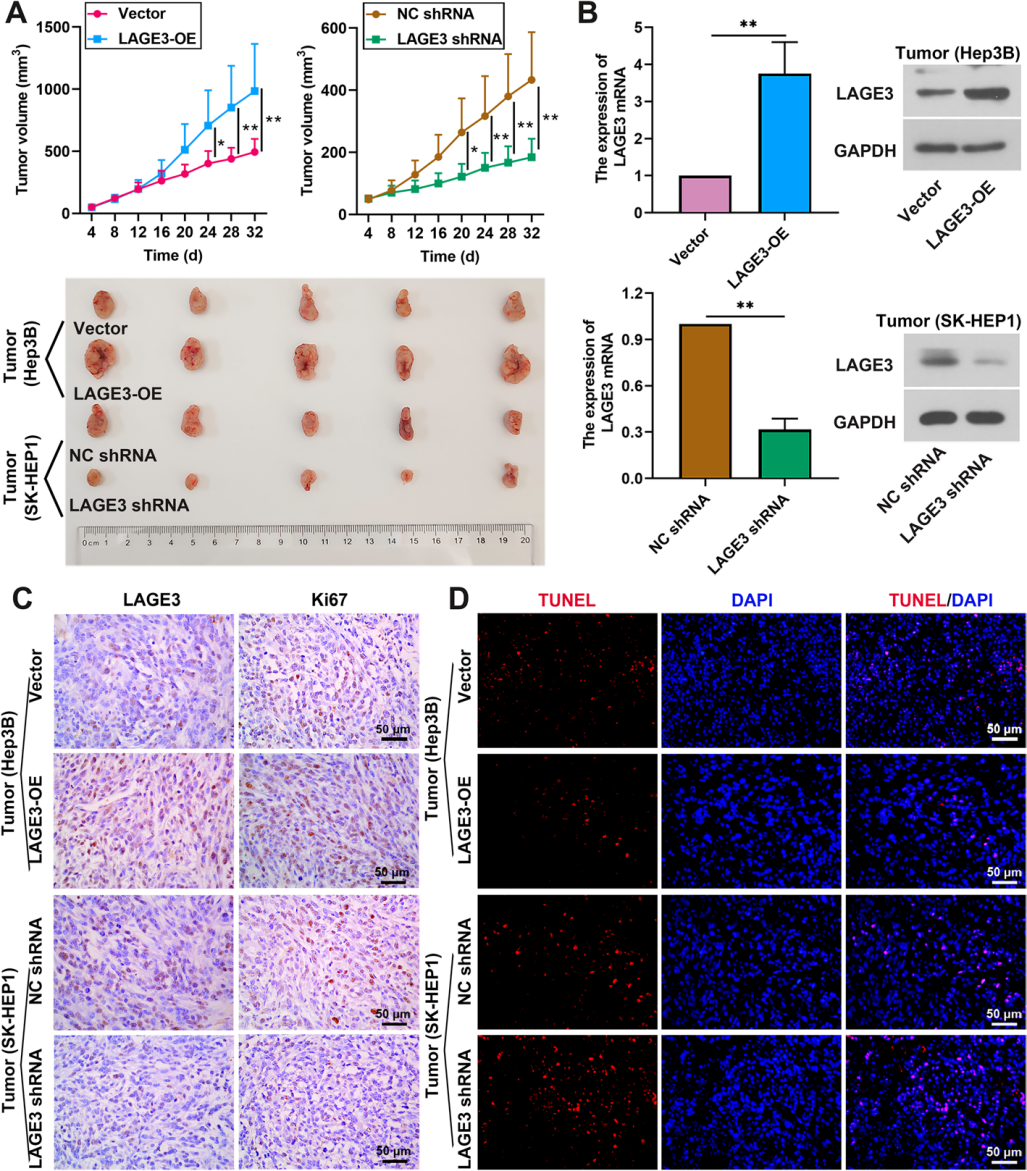
LAGE3 promoted the in vivo growth and suppressed the apoptosis of HCC tumor cells. Mice were subcutaneously injected with 2 × 10^6^ Hep3B or SK-HEP1 cells, followed by injection with 10 μg of LAGE3-OE or LAGE3 shRNA plasmid three times a week for four week. **A** Volume of tumors. **B** mRNA and protein expression of LAGE3 in tumors. **C** Expression of LAGE3 and Ki67 was evaluated by immunohistochemistry. **D** TUNEL staining of late apoptotic cells. Data are shown as mean ± standard deviation. *p < 0.05, **p < 0.01

Given the observation that LAGE3 knockdown attenuated the cell proliferation and induced the cell apoptosis in vitro, we speculated that LAGE3 might play a vital role in the in vivo growth of the HCC tumor. An HCC xenograft model was established with HCC827 cells or SK-HEP-1 cells. As shown in Fig. 4A, LAGE3 overexpression promoted growth of the HCC tumor, and LAGE3 knockdown had the opposite effect. The mRNA and protein levels of LAGE3 were increased in the tumor of mice injected with LAGE3-OE and were reduced in the tumor of mice with LAGE3 shRNA injection (Fig. 4B). Similarly, the same results of LAGE3 levels were obtained by immunohistochemistry (Fig. 4C). We also found that the LAGE3 upregulation elevated the ki67 expression of the tumors, and LAGE3 silence had contrary effects (Fig. 4C). Moreover, the gain of LAGE3 inhibited the apoptosis of tumor cells of HCC and the loss of LAGE3 facilitated the apoptosis ability of tumors. The data suggested that LAGE3 promoted the in vivo growth and suppressed the apoptosis of HCC tumors.

### LAGE3 facilitated migration and invasion of HCC cells

**Fig. 5:**
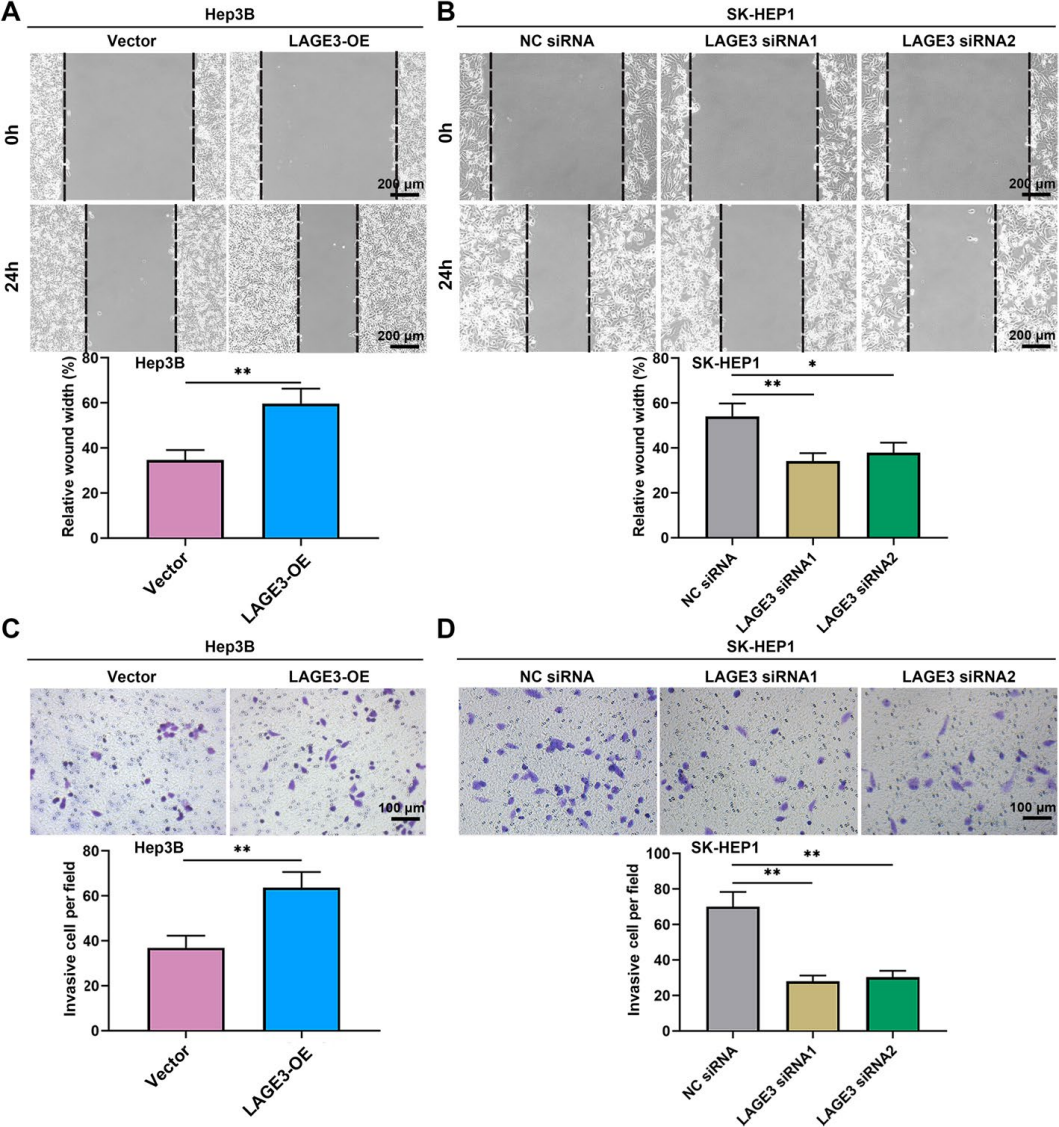
LAGE3 facilitated the migration and invasion of HCC cells. Hep-3B cells were transfected with LAGE3-OE and SK-HEP1 cells were transfected with LAGE3 siRNA1 or LAGE3 siRNA2. **A**–**B** Migration ability of HCC cells determined by wound healing assay. **C**–**D** Invasion ability of HCC cells determined by transwell assay. Data are shown as mean ± standard deviation. *p < 0.05, **p < 0.01

With the aim of clarifying the role of LAGE3 in the malignant phenotypes of HCC cells, the abilities of cell migration and invasion were measured using the wound healing assay and transwell assay. As shown in Fig. 5A and B, the upregulation of LAGE3 enhanced the migration of Hep3B cells, and the downregulation of LAGE3 led to the opposite results. Subsequently, the invasion ability was promoted by the overexpression of LAGE3 in Hep3B cells and was inhibited by the knockdown of LAGE3 in SK-HEP1 cells (Fig. 5C and D). These results indicated that LAGE3 facilitated the migration and invasion of HCC cells.

### LAGE3 aggravated HCC progression by enhancing the JNK and ERK signaling pathway

**Fig. 6:**
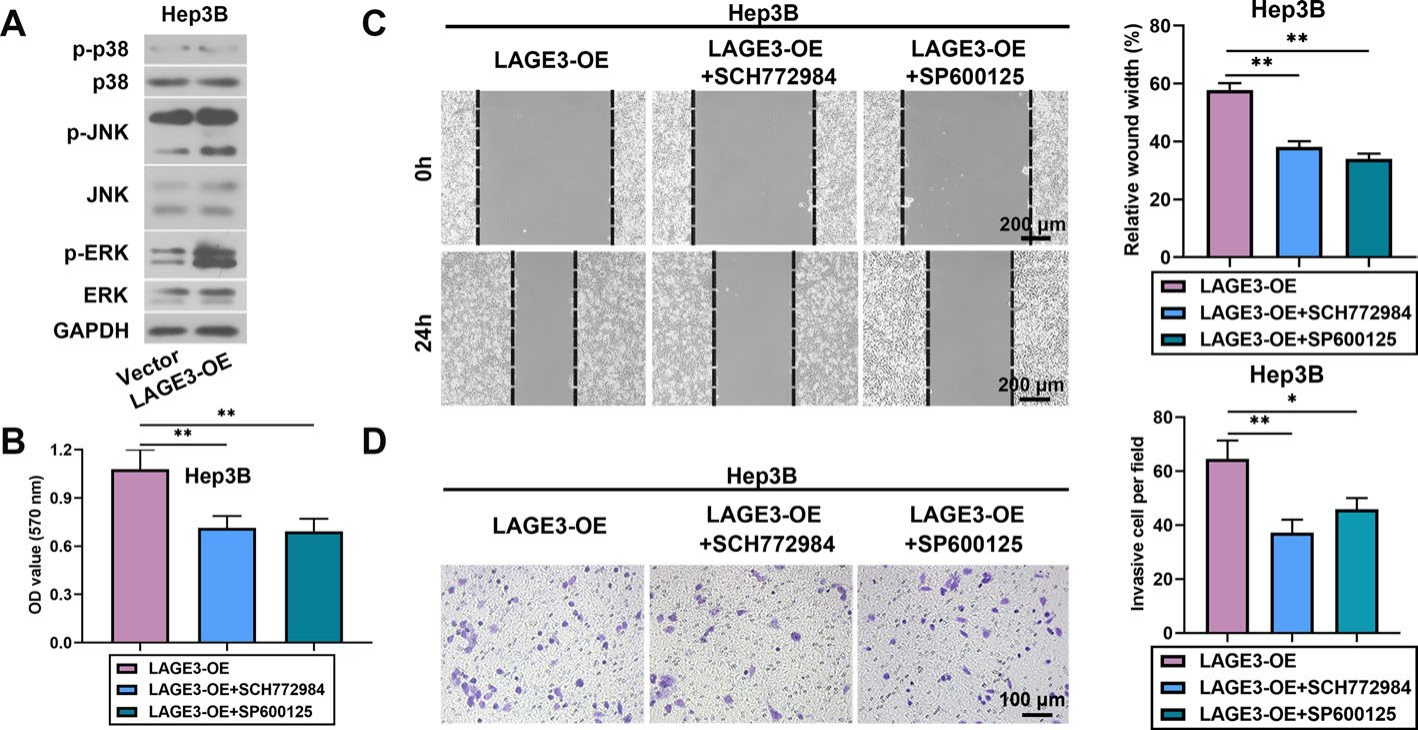
LAGE3 aggravated HCC progression by enhancing the JNK and ERK signaling pathway. Hep-3B cells were transfected with LAGE3-OE and then incubated with ERK inhibitor SCH772984 or JNK inhibitor SP600125. **A** Western blot bands of p-p38, p38, p-JNK, JNK, p-ERK, and ERK in Hep3B cells. **B** Proliferation ability of Hep3B cells. **C**–**D** Migration and invasion abilities of Hep3B cells. Data are shown as mean ± standard deviation. *p < 0.05, **p < 0.01

We next explored the underlying mechanism of the effects of LAGE3 on HCC progression. We found that the overexpression of LAGE3 increased the expression of p-JNK and p-ERK (Fig. 6A). SCH77298 or SP600125 suppressed the LAGE3 upregulation-induced proliferation, migration, and invasion of Hep3B cells, indicating that LAGE3 aggravated the HCC progression by facilitating the JNK and ERK signaling pathway. (Fig. 6B–D).

## Discussion

HCC is the second leading cause of cancer-associated death worldwide [19]. The prognosis of HCC patients remains unsatisfactory owing to the high index of tumor recurrence and metastasis [20]. Therefore, a treatment targeting the malignant phenotypes of tumor cells including cell proliferation, migration, invasion, and apoptosis is an excellent policy to alleviate the HCC progression. In the present study, LAGE3 was found to be upregulated in HCC tissues compared to normal tissues and the high expression of LAGE3 was associated with poor overall survival. LAGE3 facilitated the cell proliferation, migration, and invasion, and suppressed the apoptosis of HCC cells in vitro. These results were further confirmed by the in vivo growth of HCC xenografts and inhibition of apoptosis of the tumor cells. Also, we found that LAGE3 aggravated the HCC progression by enhancing the JNK and ERK signaling pathway. These results suggest that LAGE3 is a potential target for HCC therapy.

LAGE3 is a 14804 Da intracellular protein and a component of the complex involved in the form of N^6^-threonylcarbamoyladenosine of tRNAs [21, 22]. LAGE3 is widely expressed in various types of cells and organs in humans. Combined with the finding that LAGE3 is highly conserved and is activated in numerous human tumors, it indicates that its encoded protein is functionally pivotal in the progression of cancers [9]. LAGE3 serves as a prognostic biomarker for the clinical outcome and immune infiltration in skin cutaneous melanoma [23]. Moreover, previous studies have demonstrated that LAGE3 is highly overexpressed in various cancers. It has been reported that LAGE3 is highly expressed in breast cancer tissues compared to normal tissues and is correlated with poor prognosis as well as the adverse clinicopathological factors of patients [24]. In the current study, we found that LAGE3 was upregulated in HCC tissues compared to normal tissues and the high expression of LAGE3 was shown to be related to poor overall survival by bioinformatics analysis, which might indicate the prognostic significance of LAGE3. Moreover, researchers have found that the knockdown of LAGE3 weakens the migration and invasion abilities of breast cancer cells and decreased the epithelial-mesenchymal transition-related protein expression [17]. Goswami et al. have reported that the downregulation of LAGE3 suppresses the proliferation ability of non-small cell lung cancer cells [25]. Herein, similar results of LAGE3 were observed in HCC progression. We found that interfering in LAGE3 expression markedly repressed the HCC cell proliferation by arresting the cell cycle in G1 phase. Interestingly, LAGE3 is associated with DNA damage (the ataxia telangiectasia-mutated/ataxia telangiectasia-Rad3-related regulation of G1/S check point) by the MetaCore pathway analysis of LAGE3 co-expression genes [24]. The KEOPS complex containing LAGE3 is associated with genome maintenance [12]. The knockdown of the KEOPS genes could activate the DNA damage response, which results in cell arrest in order to allow DNA repair [12]. Thus, we speculate that LAGE3 might enhance the G1/S phase transition of cells by affecting the DNA damage response and further promote the proliferation of HCC cells. Furthermore, the downregulation of LAGE3 inhibited cell migration and invasion and induced the apoptosis of HCC cells, while the overexpression of LAGE3 promoted the malignant phenotypes of HCC. These results were further confirmed by the in vivo growth of HCC xenografts and inhibition of the apoptosis of tumor cells.

To promote a deeper understanding of LAGE3 in the HCC pathogenesis, we explored the underlying mechanism by which LAGE3 affected HCC malignant phenotypes from a molecular perspective. ERK is a member of the mitogen-activated protein kinase family, of which expression is imperative for development and its hyperactivation has a vital role in cancer progression [26]. Mitogen-activated protein kinase cascades ERK, JNK, and p38 are central signaling elements that regulate basic cellular processes such as cell proliferation, differentiation, and apoptosis in progression of various cancers including HCC [27–29]. Xue et al. have reported that the suppressor of zest 12 inhibits the migration and invasion abilities of HCC cells by suppressing the ERK1/2 signaling pathway, and ERK suppression weakens the migration and invasion of HCC cells induced by the knockdown of the suppressor of zest 12 [30]. Wang et al. have verified that the loss of JNK inhibits HCC tumor metastasis via the reduction of MMP-2, MMP-9, and N-cadherin, as well as the upregulation of E-cadherin [31]. In the current study, we found that LAGE3 promoted HCC cell proliferation, migration, and invasion, as well as inhibited cell apoptosis via facilitating the JNK and ERK signaling pathway. The ERK inhibitor or JNK inhibitor repressed the upregulated LAGE3-induced proliferation, migration, and invasion of HCC cells. Moreover, LAGE3 has been reported to interact with heat shock protein family A member 9 (HSPA9) and heat shock protein family A member 1 like (HSPA1L) by affinity purification-mass spectrometry analysis [32]. HSPA9 is a novel negative regulator of ERK signaling and may provide a target for the reactivation of tumor-suppressive signaling of the pathway in cancer [33]. The overexpression of HSPA1L is found to affect the phosphorylation of JNK in Parkinson's disease [34]. Thus, we speculate that the promoted effects of LAGE3 on ERK and JNK signaling might be linked to the interaction of LAGE3 with HSPA8 and HSPA1L. The precise mechanism whereby LAGE3 is abnormally expressed in HCC deserves further experimental verification.

At present, the function of LAGE3 is still rarely studied, especially in cancer. Only Dong et al. reported that LAGE3 is involved in the malignant progression of breast cancer [17]. We also proved the similar function of LAGE3 in HCC in the present study. In fact, the tumor pathogenesis is associated with a complex gene regulatory network. Although we confirmed the pro-tumor effects of LAGE3 in vitro and in vivo, its upstream and downstream regulation mechanism is still unclear. Studies have shown that an aberrant ubiquitination process plays an essential role in the biological processes including proteasomal degradation and transcriptional regulation of the complicated tumor microenvironment [35]. Many genes are abnormally expressed in HCC due to the aberrant ubiquitination process [36–38]. It has been reported that the cullin2 ubiquitin ligases are recruited to the KEOPS complex containing LAGE3 in the nucleus, which is involved in transcriptional regulation and threonylcarbamoyladenosine modification of tRNAs for accurate decoding of A-starting mRNA codons [39–41]. Moreover, LAGE3 is predicted to link with ubiquitin-mediated proteolysis [17]. Thus, we suspect that the high expression of LAGE3 in HCC may also be a result of abnormal ubiquitination modifications. The precise mechanism needs further investigation in the future.

## Conclusions

LAGE3 was more highly expressed in HCC tissues than adjacent tissues, and the upregulation of LAGE3 was correlated with poor overall survival of HCC patients. LAGE3 promoted HCC cell proliferation, migration, and invasion, and repressed cell apoptosis by potentiating the JNK and ERK signaling pathway.

## Data Availability

The data or materials used and/or analyzed during the current work are available from the corresponding author on reasonable request.

## Abbreviations

ERK: Extracellular signal-regulated kinase
FBS: Fetal bovine serum
HCC: Hepatocellular carcinoma
JNK: C-Jun N-terminal kinase
LAGE3: L antigen family member 3
MTT: 3-(4,5-Dimethylthiazol-2-yl)-2,5-diphenyltetrazolium bromide
PBS: Phosphate buffer saline
qRT-PCR: Quantitative real-time polymerase chain reaction
siRNAs: Short interfering RNAs
